# Milignant Pustule Communicated by a Fly

**Published:** 1883-10

**Authors:** 


### Malignant Pustule Communicated by a Fly.

The *Jour. Cut. and Ven. Dis.* for August, 1883, reports the following:

A somewhat remarkable case is reported in the *Gaz. des Hopitaux*, No. 102, for September 5th, as having occurred in the service of M. Molliere, surgeon-in-chief to the Hotel Dieu at Lyons. The patient was bitten on the cheek by a large black fly which he immediately killed. The bitten spot in a few hours began to itch violently, but no swelling appeared until the next day. When the patient entered the hospital the whole cheek was of a livid color and enormously swollen, especially over the malar bone, the center of which region was occupied by a small black phlyctena surrounded by a number of transparent vesicles. The eyelids were considerably swollen, and one of the submaxillary glands was enlarged and tender. There was no fever or other constitutional symptom. M. Molliere's treatment was prompt and energetic. He first completely destroyed the pustule by means of the thermo-cautery, and then injected the swollen parts, including the submaxillary gland, with a twenty per cent. solution of phenic acid. The only internal remedy employed was alcohol, which was administered in enormous quantities without producing the slightest sign of intoxication. The affected surface began to slough off on the third day, and in another week was entirely detached. The healing process proceeded rapidly, and at the end of three weeks the patient was discharged. Blood and serum drawn from the vicinity of the pustule having been forwarded to an eminent expert of examination, he succeeded in detecting a few filaments of the bacillus antracis, and a cobaye which was inoculated with the fluids, died in a few hours with all the signs of specific gangrenous infection.

